# Retraction: Recent advances in visible-light graphitic carbon nitride (g-C_3_N_4_) photocatalysts for chemical transformations

**DOI:** 10.1039/d4ra90095b

**Published:** 2024-09-05

**Authors:** Praveen P. Singh, Vishal Srivastava

**Affiliations:** a Department of Chemistry, United College of Engineering & Research Naini Prayagraj 211010 India ppsingh23@gmail.com; b Department of Chemistry, CMP Degree College, University of Allahabad Prayagraj 211002 India vishalgreenchem@gmail.com

## Abstract

Retraction of ‘Recent advances in visible-light graphitic carbon nitride (g-C_3_N_4_) photocatalysts for chemical transformations’ by Praveen P. Singh and Vishal Srivastava, *RSC Adv.*, 2022, **12**, 18245–18265, https://doi.org/10.1039/D2RA01797K.

The Royal Society of Chemistry, with the agreement of the authors, hereby wholly retracts this *RSC Advances* review article due to significant portions of text overlap with a number of sources throughout the review article, in particular ref. 31, 37, 67*e*, 67*j*, 167, 168, 169 and 172 of the original article and ref. 1 and 2 below. Although many of these articles have been cited, it was not made clear that some of the text was reproduced from these articles.

Signed: Praveen P. Singh and Vishal Srivastava

Date: 29th August 2024

Retraction endorsed by Laura Fisher, Executive Editor, *RSC Advances*
